# Efficacy of Pomegranate Seed Oil (PSO) in Its Nanoformulation (GranaGard^®^) in Neurodegenerative Disease Modulation

**DOI:** 10.3390/cimb48101087

**Published:** 2026-10-21

**Authors:** Kati Frid, Orli Binyamin, Liraz Larush, Shlomo Magdassi, Ruth Gabizon, Haim Ovadia

**Affiliations:** 1 Department of Neurology, The Hebrew University of Jerusalem, Jerusalem 9112001, Israel; kati.frid@gmail.com (K.F.); orli@granalix.com (O.B.); 2 Granalix Biotechnologies, Jerusalem 9342144, Israel; 3 Institute of Chemistry and Center for Nanoscience and Nanotechnology, The Hebrew University of Jerusalem, Jerusalem 9190401, Israel; liraz.larush@gmail.com (L.L.); shlomo.magdassi@mail.huji.ac.il (S.M.)

**Keywords:** Nano-PSO/GranaGard^®^, punicic acid (PA), oxidative stress, neuroprotection, neurodegenerative diseases

## Abstract

Oxidative stress contributes to the pathophysiology of diverse neurodegenerative diseases and acute brain injuries. Pomegranate Seed Oil (PSO) is rich in punicic acid (PA), an omega-5 fatty acid with reported antioxidant and anti-inflammatory properties. However, conventional PSO and standard antioxidants show limited clinical success due to poor solubility, rapid degradation, low bioavailability, and limited delivery cross the blood-brain barrier (BBB). To address these limitations, GranaGard^®^ (Nano-PSO), a self-emulsifying nanoformulation, was developed to improve the bioavailability and CNS delivery of PSO-derived compounds. Nano-PSO significantly increases systemic absorption and brain accumulation of its bioactive metabolite, neuroprotective conjugated linoleic acid (CLA). In a transgenic mouse model of Creutzfeldt-Jakob Disease (CJD), continuous Nano-PSO administration was reported to delayed disease onset, prolong survival, reduce lipid oxidation, preserve neuronal viability, and increase neurogenesis. Beyond CJD, Nano-PSO showed potential neuroprotective effects in various neurological diseases and brain injury models, although the magnitude and consistency of these effects varied across models. In a small preliminary clinical study in Multiple Sclerosis (MS), Nano-PSO treatment was associated with improved verbal learning in a within-group analysis, although large controlled studies are needed. By improving pharmacokinetic barriers, Nano-PSO may offer a promising, potent strategy for mitigating neurodegeneration associated with oxidative stress. These preliminary findings warrant further investigation, including independent replication and controlled clinical studies to establish the reproducibility and clinical relevance of Nano-PSO.

## 1. Introduction

The pomegranate (*Punica granatum* L.), a fruit native to Western Asia belonging to the Punicaceae family, has been traditionally valued since ancient times for both its nutritional and potential medicinal properties [1]. Notably, it is referenced in the biblical Song of Songs as a symbol of love, beauty, and fertility, highlighting its long-standing cultural significance. In modern research, pomegranate is widely recognized as a “superfood” due to its rich content of polyphenols—particularly ellagitannins (e.g., punicalagins) and anthocyanins—which exhibit high levels of antioxidant and anti-inflammatory activity [2]. Various parts of the fruit, including the peel (rind), seeds, flowers, bark, and roots, have been traditionally used either directly or as extracts to treat a wide range of conditions, including gastrointestinal disorders, wound healing, and skin irritation. Bark and root extracts have been specifically utilized to eliminate intestinal parasites [3].

Pomegranate Seed Oil (PSO) is distinguished by its high content of punicic acid (PA), an omega-5 conjugated α-linolenic acid (CLnA) isomer that comprises up to 80% of its composition [4,5]. PA is associated with strong antioxidant, anti-inflammatory, metabolic, and antiproliferative properties [4,5,6,7].

Oxidative stress is an important contributing factor in the pathogenesis of multiple neurodegenerative diseases, including Alzheimer’s disease (AD) [8,9], Parkinson’s disease (PD) [10,11], multiple sclerosis (MS) [12,13], and Creutzfeldt-Jakob disease (CJD) [14,15], as well as acute neurological injuries such as traumatic brain injury (TBI) [16,17] and cerebral ischemia-reperfusion injury [18,19]. Growing evidence indicates that oxidative stress contributes to disease initiation and progression across these pathologies [20]. This effect is largely mediated by the excessive production of reactive oxygen species (ROS), which induce oxidative damage to lipids, proteins, and nucleic acids, ultimately leading to neuronal dysfunction and cell death [21].

Despite extensive investigation, conventional antioxidant therapies have shown limited clinical efficacy. This limitation is attributed not only to poor stability and insufficient bioavailability but also to restricted penetration across biological barriers, including the blood-brain barrier (BBB) [22], and limited delivery to relevant intracellular compartments such as mitochondria [23]. These challenges highlight a critical gap between the antioxidant potential observed in vitro and the relatively low therapeutic efficacy achieved in clinical settings [22].

In this context, nanoformulation strategies may help address some of these challenges. Self-emulsifying delivery systems can improve the solubility, stability, and absorption of lipophilic compounds such as PSO and its major bioactive component PA [4,7,24]. Such formulations may enhance tissue distribution and facilitate penetration across biological barriers, including the BBB, potentially improving delivery to both central and peripheral target tissues to better counteract oxidative stress [22,25].

Among these strategies, the nanoformulated PSO product commercially known as GranaGard^®^ (Nano-PSO) has been investigated in several preclinical studies and preliminary clinical research. Findings from both our laboratory and independent studies suggest that nanoformulated PSO demonstrates improved bioavailability, enhanced delivery to the central nervous system (CNS), and significant neuroprotective effects across multiple experimental models [26,27,28,29,30]. Notably, PSO, particularly in its nanoformulated form, not only exhibits antioxidant activity through direct free radical scavenging but also modulates mitochondrial function [28,31], reduces lipid peroxidation [32], attenuates neuroinflammation, and preserves neuronal viability [28,33,34].

This review aims to critically evaluate the therapeutic potential of pomegranate seed oil, with a particular focus on its nanoformulation (GranaGard^®^), by summarizing current evidence regarding its antioxidant and neuroprotective effects, as well as its potential to improve bioavailability and tissue delivery in diseases where oxidative stress plays a central role, while acknowledging the limitations of the available evidence.

## 2. Pomegranate Seed Oil (PSO) and Punicic Acid: Composition, Bioactive Properties, and Antioxidant Mechanisms

Pomegranate seed oil (PSO) represents approximately 12–20% of the total seed weight and contains 14 fatty acids. Its predominant fatty acid is punicic acid (PA), which comprises approximately 50–80% of the total fatty acid content, followed by linoleic acid (13–20%), oleic acid (8–9%), palmitic acid (6–9%), stearic acid (2–3%), linolenic acid (0.06–0.08%), and arachidic acid (0.68–0.90%) [4,25]. While the chemical composition of PSO may vary depending on cultivar, geographical origin, extraction method, and storage conditions, PSO in GranaGard^®^ mostly comprises 80–90% of PA.

PA is an omega-5 fatty acid and a conjugated α-linolenic acid (CLnA) isomer characterized by three conjugated double bonds in a *cis*-9, *trans*-11, *cis*-13 configuration [24,25]. This unique conjugated structure is believed to contribute significantly to the antioxidant properties of PA and PSO [35].

PSO has shown free radical-scavenging in cell-free assays, including DPPH and ABTS tests [36,37,38]. Additionally, PSO presents antioxidant and cytoprotective effects in cultured cells [37], while PA itself has been reported to reduce oxidative stress and improve antioxidant defense parameters in experimental models [35,39]. Together, these findings suggest that both PSO and PA in particular may contribute to protection against oxidative damage through antioxidant and cellular protective mechanisms, although these findings do not directly establish equivalent activity of Nano-PSO.

In addition to direct antioxidant activity, PA modulates intracellular signaling pathways involved in lipid metabolism, inflammation, and redox regulation. PA has been shown to activate peroxisome proliferator-activated receptors (PPARs), including PPAR-α and PPAR-γ, these transcription factors regulate glucose homeostasis and inflammatory responses, and experimental studies have demonstrated that their activation improves glucose tolerance and reduces obesity-associated inflammation [25,40]. Consistent with these findings, Nano-PSO improved glucose tolerance and insulin sensitivity and reduced hepatic steatosis in mice fed a high-fat diet. These effects were associated with increased fatty acid oxidation in primary hepatocytes and elevated expression of Nrf2 and PPAR-related genes [41].

Beyond these metabolic actions, PA has also demonstrated antiproliferative effects [5], cardiovascular protective qualities (including improved lipid profiles and reduced vascular oxidative stress) [7], and the capacity to enhance endogenous antioxidant enzyme systems such as superoxide dismutase (SOD), catalase (CAT) and glutathione peroxidase (GPx) [25,35,37]. Collectively, these findings point to a broad and multifaceted biological profile that extending well beyond the role of PA as a direct radical scavenger.

The pharmacological activity of PSO, however, is unlikely to be mediated by PA alone; other bioactive constituents, including phytosterols and polyphenolic compounds, may also contribute to its overall effects [2,6,24]. Taken together, PSO appears to act through multiple complementary mechanisms, positioning it as a bioactive oil whose therapeutic potential extends beyond its classical role as an antioxidant [2,6,7].

Nevertheless, translating these properties into therapeutic benefits for neurological disorders requires more than biological activity alone. PA can be metabolically converted into the conjugated linoleic acid (CLA) isomer 9c,11t-CLA, a metabolite with established relevance to neuronal protection [27]. Achieving sufficient brain delivery of this bioactive metabolite remains a critical challenge in the neurological application of PSO. Nano-PSO was developed to improve the bioavailability of PSO-derived compounds. Consistent with this rationale, comparative studies demonstrated significantly higher brain levels of 9c,11t-CLA following Nano-PSO administration compared with conventional PSO [27]. However, direct radical-scavenging activity of the finished nanoformulation has not been established.

### Oil Characterization and Standardization

PSO was extracted from the fruit seeds by cold pressing (Gan Shmuel Group, Gan Shmuel, Israel). Batches were analyzed for the content of fatty acids by gas chromatography and found to contain 80–90% punicic acid (Milouda (Mérieux NutriSciences), Acre, Israel). Other physical characteristics were as follows: acid value 0.61% *w*/*w*, relative density 0.94 at 20 °C, and peroxide value 1.5 meq O_2_/kg. The oil was kept under nitrogen to avoid exposure to atmospheric oxygen until use. The generation of the Nano-PSO self-emulsifying formulation (GranaGard^®^) with an average particle size of 200 nm was produced as described in US Patent Application No. 14/523,408. The commertial form of GranaGard^®^ is performed in Ambrosia Supherb Ltd., Nof HaGalil, Israel.

## 3. Oxidative Stress as a Common Denominator in Neurodegenerative Diseases

Oxidative stress is defined as an imbalance between the production of reactive oxygen species (ROS) and the capacity of endogenous antioxidant defense systems to neutralize them [20,21]. This imbalance is recognized as a significant contributor to the pathogenesis of multiple neurodegenerative diseases [20,21]. Although each disease is characterized by distinct pathological hallmarks, accumulating evidence consistently identifies oxidative damage as a central event in the progression of neurodegeneration [13,14,20].

In Alzheimer’s disease (AD), oxidative damage is widely considered an early pathogenic event rather than a mere downstream consequence [20,42]. Elevated levels of lipid peroxidation products, including F2-isoprostanes and 4-hydroxynonenal, and nucleic acid oxidation markers such as 8-hydroxy-2-deoxyguanosine (8-OHdG), have been consistently detected in the brains and cerebrospinal fluid of AD patients, including individuals at early clinical stages such as mild cognitive impairment (MCI) [8,9,42]. Amyloid-beta (Aβ) can interact with copper and iron ions, which participate in redox reactions and promote ROS generation, inflicting further oxidative damage on nearby proteins, lipids, and nucleic acids [8]. Concurrently, hyperphosphorylated tau and neurofibrillary tangle formation have been linked to mitochondrial dysfunction and elevated ROS generation [9]. Together, these pathways create a self-reinforcing cycle of disease progression in which oxidative stress promotes pathological protein accumulation, leading to amplified oxidative damage [8,9].

In Parkinson’s disease (PD), the selective degeneration of dopaminergic neurons in the substantia nigra is strongly associated with oxidative stress [10,11]. The spontaneous oxidation of dopamine and its metabolism by monoamine oxidase (MAO) generate ROS, including hydrogen peroxide and superoxide, while Complex I deficiency in the mitochondrial electron transport chain further amplifies this burden [10,11]. Iron accumulation in the substantia nigra may contribute to ROS production and increase oxidative stress [43], and the aggregation of α-synuclein into Lewy bodies contributes to this damage, as demonstrated by the widespread presence of nitrated α-synuclein within these inclusions [44].

In Multiple Sclerosis (MS), oxidative stress is a key pathological feature that contributes to inflammation, demyelination, and axonal damage within MS lesions [12,13,20]. Activated microglia and macrophages generate high levels of ROS and reactive nitrogen species (RNS), including peroxynitrite, which cause oxidative damage to myelin sheaths [12,13]. Because myelin has a high lipid content, it is particularly vulnerable to lipid peroxidation. Consistent with this, increased urinary levels of lipid peroxidation markers have been reported in MS patients [45]. Furthermore, oxidative damage to axonal mitochondria has been implicated in the irreversible neurodegenerative processes associated with progressive MS [13].

In prion diseases such as Creutzfeldt-Jakob disease (CJD), the neurotoxicity associated with the accumulation of misfolded prion protein (PrP) involves oxidative pathways [14,46]. Misfolded PrP disrupts copper and iron homeostasis, thereby promoting the generation of highly reactive hydroxyl radicals [46]. Correspondingly, lipid peroxidation markers such as malondialdehyde (MDA) have been detected in the cerebrospinal fluid and serum of CJD patients [14]. Additionally, activated microglia upregulate NADPH oxidase (NOX2), which promotes increased ROS production in affected brain regions [46], while mitochondrial dysfunction further contributes to the oxidative burden as an early and central component of prion disease pathogenesis [31]. Furthermore, the dysregulation of redox-active metals such as iron, copper, zinc, and manganese through altered divalent metal transporter expression represents an early hallmark of prion disease, amplifying oxidative stress before signs of neurodegeneration become clinically evident [46].

In traumatic brain injury (TBI) and cerebral ischemia-reperfusion injury (CIRI), oxidative stress plays a central role in neuronal damage by activating cellular pathways similar to those described in chronic neurodegenerative diseases [17,18,19]. In TBI, the initial injury increases ROS production, leading to lipid peroxidation, neuroinflammation, and synaptic dysfunction In CIRI, ischemia promotes free radical production, while reperfusion causes a rapid ROS burst that leads to mitochondrial damage and neuronal apoptosis [18,19]. Together, these findings highlight oxidative stress as a shared mechanism of injury in both TBI and CIRI, positioning it as a promising therapeutic target for acute neurological injury.

Overall, oxidative stress emerges as a shared pathological mechanism across both neurodegenerative diseases and acute neurological injuries, providing a strong rationale for antioxidant-based therapeutic strategies [20,21].

## 4. Limitations of Conventional Antioxidant Therapies

Because oxidative stress plays a central role in the pathogenesis of many neurodegenerative diseases and neurological injuries, antioxidant-based treatments have been widely explored as a potential therapeutic approach [21,47] (DASH, LIU). However, although these treatments can support the antioxidant defense system by neutralizing ROS and reducing oxidative damage, many clinical trials have failed to show meaningful therapeutic benefits [48]. This gap can be explained by several factors.

The first relates to the pharmacokinetic properties of antioxidant compounds. Many possess poor aqueous solubility and chemical instability, leading to rapid degradation before reaching target tissues; oral administration further reduces their bioavailability and limits accumulation at the site of action [22,48]. The second concerns tissue and subcellular distribution. In neurodegenerative diseases and neurological injuries where the brain is the primary target, many antioxidants have a limited ability to cross the BBB, reducing their potential to reach the CNS [22]. Even antioxidants that do reach the brain may fail to accumulate sufficiently within mitochondria—the primary intracellular source of ROS—further limiting their protective efficacy [23]. The third relates to specificity. Most conventional antioxidants act non-selectively and may interfere with physiological ROS signaling essential for normal cellular function [47].

Together, these pharmacokinetic, distributional, and mechanistic limitations help explain why the antioxidant potential observed in preclinical studies does not always translate into clinical success. Improving antioxidant stability, bioavailability, BBB penetration, and mitochondrial targeting is therefore essential to unlock their therapeutic potential in neurodegenerative diseases.

## 5. Nanoformulation Strategies for Lipophilic Bioactive Compounds

Nanoformulation strategies have been developed to improve the delivery of poorly water-soluble and chemically unstable bioactive compounds [22]. By reducing droplet size and increasing surface area, these approaches enhance the dispersion of lipophilic compounds in aqueous gastrointestinal fluids, protect them against degradation, and improve intestinal absorption and tissue distribution [22,49].

### 5.1. Self-Emulsifying Delivery Systems (SEEDS)

Nano-PSO (GranaGard^®^) falls under the pharmacological category of self-emulsifying delivery systems (SEEDS). SEEDS represent a well-established and industrially feasible approach in pharmaceutical formulation to overcome the inherently low oral bioavailability associated with hydrophobic, lipophilic drugs and bioactive oils. These formulations consist of an isotropic mixture of natural or synthetic oils, solid or liquid surfactants, and co-solvents. Upon contact with gastrointestinal fluids and gentle agitation provided by digestive motility, SEEDS spontaneously emulsify to form fine oil-in-water dispersions. This rapid dispersion dramatically expands the surface area available for enzymatic digestion and absorption across the intestinal mucosa [49].

### 5.2. Optimizing Safety, Droplet Mechanics, and Dosage

Because Nano-PSO (GranaGard^®^) is intended for use as a functional food supplement, specific formulation considerations are paramount. Factors such as the toxicity and safety of surfactant excipients, alongside the size and surface charge of the generated oil droplets, are crucial for both health/tolerability and gastrointestinal absorption efficiency [50,51]. By leveraging proven pharmaceutical SEEDS technology:

- Enhanced Bioavailability: The formulation overcomes the poor water solubility and limited absorption of conventional oil administration [26,50].
- Lower Effective Dosage: It justifies the use of significantly lower overall dosages of PSO to achieve maximal biological effects compared to raw oil.
- Active Metabolite availability: Improved dispersion may protect punicic acid (PA) during digestion, and thereby facilitate its systemic availability [26,50].

Following oral administration, PA is rapidly metabolized in the liver into the conjugated linoleic acid (CLA) isomer 9c,11t-CLA, a compound with demonstrated neuroprotective properties [27]. In mouse models, this targeted delivery allows CLA to accumulate in the brain following Nano-PSO administration, whereas a comparable accumulation is not detected after administering conventional, unformulated PSO [27,52]. Thus, nanoformulation via SEEDS fundamentally alters both the pharmacokinetic profile and tissue distribution of PSO-derived bioactive metabolites.

## 6. Neuroprotective Effects of Nano-PSO (GranaGard^®^) Across Disease Models

The neuroprotective effects of nanoformulated pomegranate seed oil, commercially known as Nano-PSO, have been investigated in several neurological conditions. Evidence from preclinical animal models supports its potential therapeutic effects in prion disease, Alzheimer’s disease, Parkinson’s disease, multiple sclerosis, traumatic brain injury, and cerebral ischemia/reperfusion injury, with additional preliminary clinical evidence from a pilot study in MS. The following subsections summarize the main findings obtained in each condition and discuss the mechanisms that may contribute to the neuroprotective effects of Nano-PSO (Table 1).

### 6.1. Efficacy of Nano-PSO (GranaGard^®^) in Prion Disease (Creutzfeldt-Jakob Disease)

Creutzfeldt–Jakob disease (CJD) is a fatal human prion disease characterized by a long asymptomatic period followed by progressive neurological decline and the accumulation of abnormal prion protein in the central nervous system, with no effective therapy currently available [57]. TgMHu2ME199K mice a transgenic model of genetic CJD associated with the E200K PrP mutation, was used to evaluate the neuroprotective potential of Nano-PSO [26,32]. These studies demonstrated that Nano-PSO delayed disease progression when treatment was initiated during either the asymptomatic or symptomatic stage [26]. Long-term continuous administration also prolonged survival, with 50% of Nano-PSO-treated mice remaining free of terminal-stage disease at 18 months of age, whereas all untreated mice had already reached this endpoint [32]. However, maintenance of this state required continuous treatment, as discontinuation of Nano-PSO at 9 months led to rapid deterioration within six weeks, with disease scores quickly approaching those of untreated mice [32].

The clinical benefits observed across these studies were associated with reduced lipid oxidation and neuronal loss, preservation of synaptophysin expression, and increased hippocampal neurogenesis [26]. In the long-term study, Nano-PSO treatment was additionally associated with reduced glycosaminoglycan (GAG) accumulation in the brain, retina, and urine [32]. Surprisingly, there was no reduction in the accumulation of disease-related or proteinase K-resistant PrP suggesting that the neuroprotective effects of Nano-PSO may protect the CNS from pathological damage through mechanisms that do not directly depend on reducing PrP accumulation.

### 6.2. Anti-Aging Effects of Nano-PSO (GranaGard^®^)

Metformin, an anti -diabetic drug, was shown to extend longevity [58], and to ameliorate the activity of recognized aging hallmarks [59]. Such hallmarks are mostly related to antioxidant and anti-inflammatory pathways. To this effect, we compared the effect of GranaGard^®^ and metformin on the levels of such markers in TgMHu2ME199K mice. Indeed, both treatments produced similar effects on several aging-related molecular and cellular markers, including AMPK, COX IV-1, Nrf2 and HIF-1α, IGF-1, inflammatory markers, and endogenous neural stem cells generation. However, only Nano-PSO delayed clinical disease progression [55], suggesting that modulation of these shared pathways alone may not fully explain its therapeutic effect.

### 6.3. Efficacy of Nano-PSO (GranaGard^®^) in Alzheimer’s Disease

Alzheimer’s disease (AD) is characterized by progressive cognitive decline, amyloid-beta (Aβ) accumulation, tau hyperphosphorylation and aggregation, mitochondrial dysfunction, and oxidative stress [60]. The efficacy of Nano-PSO was evaluated in the 5XFAD mouse model, which carries five familial AD mutations [61]. Nano-PSO was administered continuously in drinking water from three weeks of age until 10 months of age [27].

Long-term treatment with Nano-PSO preserved spatial memory and habituation, as assessed by the T-maze and open field habituation tests. These mice also tended to perform better in the novel object recognition test, suggesting an improvement in recognition memory. Interestingly, Nano-PSO-treated wild-type mice also showed improved T-maze performance, suggesting that its cognitive effects may extend beyond the AD model and provide protection against age-related cognitive decline [27].

Pathologically, Nano-PSO treatment reduced intracellular and extracellular Aβ accumulation and amyloid plaque burden, and restored the expression of the mitochondrial protein COX IV-1 [27], a potential marker of mitochondrial dysfunction in neurodegenerative disease [62]. This aligns with previous studies showing that Aβ can accumulate within mitochondria [63,64]. Treatment also increased brain levels of the isomer 9c,11t-CLA—an effect that was not observed following the administration of PSO in its native form [27]—consistent with the conversion of punicic acid to CLA [7].

In addition, Nano-PSO reduced p25 levels [27], suggesting possible modulation of the calpain-p25-CDK5 pathway, whose role is well established in neurodegenerative diseases [65,66]. This explanation is supported by in vitro findings showing that CLA acts as a μ-calpain-specific inhibitor that reduces Aβ and p25 accumulation [67], although calpain activity itself was not directly assessed, and its involvement therefore remains hypothetical [27].

### 6.4. Efficacy of Nano-PSO (GranaGard^®^) in Multiple Sclerosis

The efficacy of Nano-PSO was first evaluated in a mouse model of experimental autoimmune encephalomyelitis (EAE), the most widely used animal model of multiple sclerosis (MS) [29]. MS is a chronic immune-mediated disease of the CNS characterized by demyelination, inflammation, axonal damage, and increased oxidative stress, leading to progressive neurological dysfunction [68,69,70]. The EAE model recapitulates several of these key pathological features [71].

Nano-PSO significantly reduced clinical disease severity at doses equivalent to just 1% of the amount of native PSO required to produce a similar effect. This effect was directly attributed to punicic acid, as a comparable Nano-Soybean formulation lacking punicic acid showed no beneficial effect [29]. Importantly, the therapeutic effect depended on droplet size: the 180 nm formulation was highly effective, whereas a smaller 30 nm formulation was ineffective, so efficacy cannot be assumed across nanoformulations [29].

Nano-PSO also reduced disease severity when treatment was initiated after disease onset, though the therapeutic effect depended heavily on the timing of administration. Treatment from the day of EAE induction postponed the appearance of clinical symptoms and reduced disease severity, whereas treatment initiated 7 days after induction improved clinical outcomes but did not alter the time course of disease development [29]. These findings suggest that Nano-PSO can be beneficial even when early signs of demyelinating disease are already established. Pathologically, Nano-PSO reduced both demyelination and the accumulation of oxidized lipids in EAE mice [29], mirroring evidence of elevated lipid peroxidation in MS patients [45]. The marked effects reported in EAE require replication under comparable conditions in additional laboratories. Because EAE is an immune-mediated model, peripheral immunomodulatory effects may contribute to clinical improvement.

Based on these results, Nano-PSO was subsequently evaluated in a small randomized, double-blind, crossover pilot study in 30 patients with MS, of whom 22 completed the study. One group received Nano-PSO for 3 months followed by a placebo for 3 months, while the second group received the treatments in reverse order. All patients were concurrently receiving immunomodulatory therapy [54]. No significant between-group difference in EDSS was observed. In the main within-group analysis, three months of treatment significantly improved verbal learning (CVLT-II) and overall cognitive z-scores compared to baseline. Specifically, the mean CVLT-II z-score increased from 0.891 to 1.415 (*p* = 0.012), while the mean overall cognitive z-score increased from −0.0077 to 0.387 (*p* = 0.034). However, in a mixed-effects model for repeated measures (MMRM) comparing the treatments while adjusting for potential carryover effects, the CVLT-II result remained statistically significant (*p* = 0.05), whereas the improvement in the overall cognitive z-score did not (*p* = 0.09). Importantly, no significant changes occurred in the placebo group during the same period, although this alone does not establish a significant treatment–placebo difference. A similar but non-significant trend was observed in the brief visuospatial memory test (BVMT-R), whereas no change was found in the Symbol Digit Modalities Test (SDMT) [54]. The observed cognitive improvements appeared to persist during the subsequent three months period, although this observation may be influenced by carryover and practice effects. However, the study has notable limitations, including its small sample size (22 completers out of 30 enrolled participants) and short duration. Repeated cognitive testing may introduce practice effects, while potential carryover effects should also be considered in the crossover design. Furthermore, confidence intervals and standardized effect sizes were not reported, and the observed changes were not evaluated against established thresholds for clinically meaningful improvement. Therefore, the clinical relevance of these findings remains uncertain. Larger studies with longer follow-up periods are needed to confirm these initial observations.

### 6.5. Efficacy of Nano-PSO (GranaGard^®^) in Parkinson’s Disease

Parkinson’s Disease (PD) is a progressive neurodegenerative disorder characterized by the degeneration of dopaminergic neurons in the substantia nigra and the accumulation of aggregated α-synuclein, driven largely by mitochondrial dysfunction and oxidative stress [72,73]. The effects of Nano-PSO were evaluated in a rotenone-induced rat model of parkinsonism, where rotenone inhibits mitochondrial complex I, leading to oxidative stress, dopaminergic neuronal damage, impaired dopamine signaling, α-synuclein alterations, and motor dysfunction [56,74].

Nano-PSO was administered either before or after rotenone exposure to evaluate its ability to prevent or reverse rotenone-induced neurological damage. Treatment produced moderate improvements in motor, neuronal, dopaminergic, oxidative, and transcriptional parameters. Preventive treatment generally showed greater effects on motor performance, dopaminergic neuron preservation, and lipid peroxidation, whereas treatment after rotenone exposure showed stronger effects on certain dopaminergic and transcriptional profiles [56].

However, responses varied considerably among individual animals, and across the assessed outcomes, with improvements in some measures not consistently accompanied by improvements in others and Nano-PSO did not consistently restore all parameters to normal levels. These findings suggest that Nano-PSO provides partial and phenotype-dependent protection against rotenone-induced neurodegeneration, while highlighting variability in individual treatment responses [56].

These findings should be interpreted cautiously, as they are based on a single rotenone-induced rat model and have not yet been replicated in genetic models of Parkinson’s disease or evaluated clinically. The marked inter-animal variability further suggests that treatment responsiveness may depend on the individual phenotype and experimental context. Moreover, the lack of long-term follow-up and systematic dose-response assessment limits the interpretation of the findings. Additional studies are required to determine the reproducibility and generalizability of these effects.

### 6.6. Efficacy of Nano-PSO (GranaGard^®^) in Acute Brain Injury

Beyond models of chronic neurodegeneration, Nano-PSO has shown neuroprotective effects in two models of acute brain injury: traumatic brain injury (TBI) and ischemic stroke.

In a mouse model of TBI, Nano-PSO was administered either preventively before injury or therapeutically after injury [28]. In both schedules, Nano-PSO reduced deficits in visual and spatial memory, as assessed by the novel object recognition and Y-maze tests [28]. These cognitive benefits were associated with a significant reduction in neuronal loss in the cortex and hippocampus, decreased astrogliosis, and restored mitochondrial COX IV-1 expression [28], suggesting changes in mitochondrial-related processes, although mitochondrial function was not directly assessed in this model. In mice treated post-injury, Nano-PSO also elevated cortical levels of SIRT1 and synaptophysin, markers of synaptic integrity and cell survival [28].

Similar effects were observed in a rat model of cerebral ischemia-reperfusion injury. Nano-PSO administration before or after ischemia improved locomotor recovery and reduced spatial working memory deficits in the T-maze test [30]. The findings also showed a reduction in BBB disruption, hippocampal neuronal loss in the CA1 and CA3 regions, and astrogliosis, while improving markers of oxidative stress, including lipid peroxidation and nitric oxide metabolites [30,75].

In both acute injury models, treatment administered before the injury provided greater protection than treatment administered after [28,30].

### 6.7. Combined Treatment with Nano-PSO (GranaGard^®^) and Stem Cell Transplantation

Stem cell transplantation is a promising approach for treating neurodegenerative diseases, but the poor long-term survival of transplanted cells within hostile, oxidative, and inflammatory central nervous system (CNS) microenvironments often curtails their therapeutic efficacy [76,77]. Studies combining Nano-PSO with stem cell transplantation in both Creutzfeldt-Jakob disease (CJD) and experimental autoimmune encephalomyelitis (EAE) models demonstrate that Nano-PSO significantly prolongs graft survival, thereby extending and enhancing their neuroprotective and immunomodulatory effects [29,33,34].

- CJD Model (TgMHu2ME199K mice): While neural precursor cell (NPC) transplantation alone in symptomatic mice arrested disease progression for only 30–40 days before clinical scores deteriorated, combining it with Nano-PSO treatment extended this period of disease arrest to 70–80 days and prolonged overall survival. NPCs transplanted alone quickly displayed markers of cell death and lost stem cell marker expression, whereas those co-treated with Nano-PSO remained viable and migratory for much longer [33,34].
- EAE Model of Multiple Sclerosis: Human mesenchymal stem cell (hMSC) transplantation alone reduced disease severity during the initial phase of EAE, but this protection declined after approximately 20 days and vanished following a MOG booster-induced relapse. In contrast, combining hMSC transplantation with Nano-PSO maintained clinical protection during both disease phases. This correlated directly with cell survival; hMSCs (identified via a human-specific mitochondrial marker) were still detected in the brain 60 days post-transplantation only in animals that received the combined treatment [53].

Across both experimental models, the combined treatment consistently yielded a greater increase in endogenous neurogenesis and a more pronounced reduction in astrogliosis than either Nano-PSO or stem cell transplantation alone [34,53]. Ultimately, these findings indicate that Nano-PSO improves the survival, migration, and therapeutic activity of transplanted cells, potentially through modulation of the oxidative and inflammatory environment of the diseased brain [76,77].

## 7. Mechanistic Insights into Nano-PSO (GranaGard^®^) Activity

The protective activity of Nano-PSO appears to involve the modulation of several biological processes rather than by a single molecular pathway or disease-specific pathology. Studies across models of prion disease, Alzheimer’s disease, multiple sclerosis, Parkinson’s disease, traumatic brain injury, and ischemia-reperfusion injury consistently show that Nano-PSO affects processes common to both chronic neurodegeneration and acute neuronal injury. These include oxidative stress, mitochondrial dysfunction, neuroinflammation, and impaired neurogenesis.

### 7.1. Pharmacokinetic Basis: Nanoformulation and Brain Delivery

Several studies suggest that the therapeutic effect of PSO depends fundamentally on the nanoformulation used for its delivery. In the EAE model, relatively high daily doses of natural PSO given in food (100–300 µL) produced only a moderate reduction in disease burden, whereas Nano-PSO given by gavage at a much lower dose (0.8 µL/day) almost completely prevented disease presentation [29]. This suggests that the nanoformulation significantly improves the biological activity of PSO.

The physical dimensions of the nanoformulation also appear to be critical. In the same model, the 180 nm formulation was clinically effective, whereas an equivalent dose of 30 nm droplets showed no beneficial effects [29]. A similar observation was reported in TgMHu2ME199K mice, where continuous administration of a self-emulsifying Nano-PSO formulation with a comparable droplet size (~181 nm) produced significant clinical benefit, whereas a Nano-Soybean oil formulation with the same droplet size did not [32].

The specific droplet size of the nanoformulation likely improves the bioavailability of PSO-derived active metabolites, potentially by protecting them from early hepatic metabolism and enhancing their ability to cross the BBB to reach the brain parenchyma [29,32].

### 7.2. Antioxidant Action and Mitochondrial Protection

Across different disease models, the reduction of oxidative damage and preservation of mitochondrial function appear to be central to the protective effect of Nano-PSO. In the EAE model, Nano-PSO substantially reduced the accumulation of oxidized phospholipids in the brain, as shown by EO6 immunostaining, and lowered malondialdehyde (MDA) levels close to those observed in naïve mice [29]. Similar antioxidant effects were observed in the cerebral ischemia-reperfusion model, where Nano-PSO treatment (whether given before or after injury) reduced MDA and nitric oxide metabolites while increasing total superoxide dismutase (SOD) activity [30]. In the same model, pre-treatment reduced Evans blue extravasation, suggesting preservation of blood-brain barrier integrity [30]. Reduced lipid oxidation was also reported in the CJD mouse model [32].

In a rotenone-induced model of parkinsonism [74], Nano-PSO shifted lipid peroxidation and several metabolic and transcriptional alterations back toward values observed in intact animals, though the effects varied among individuals [56].

In parallel, Nano-PSO was associated with changes in mitochondrial-related markers and functional parameters, depending on the experimental model. COX IV-1 expression, which was reduced in several disease models, was restored following Nano-PSO administration in CJD, AD, and TBI models [27,28,55]. In the CJD model, direct measurements of mitochondrial respiratory-chain enzymatic activities, ATP production, and ROS production provided additional evidence of changes in mitochondrial function [53], whereas COX IV-1 expression alone does not establish functional recovery in the other models [31]. In the TBI model, Nano-PSO also prevented the reduction of SIRT1—a NAD dependent deacetylase involved in stress response and synaptic plasticity—and increased the expression of synaptophysin (SYP), suggesting additional effects on synaptic preservation [28].

A direct comparison with metformin indicates that the modulation of shared antioxidant, mitochondrial, inflammatory, and aging-related pathways alone is not sufficient to explain the therapeutic benefit of Nano-PSO. Although both treatments affected AMPK, COX IV-1, Nrf2, IGF-1, inflammatory markers, and endogenous neural stem cell generation, only Nano-PSO delayed clinical disease progression [55]. Nano-PSO also reduced GAG accumulation and increased the expression of lysosomal enzymes involved in GAG degradation, effects that were not observed with metformin [55]. However, the reduction in oxidative-damage markers observed in vivo does not establish direct radical-scavenging activity of Nano-PSO, as these effects may also reflect changes in metabolism, inflammation, or disease progression.

### 7.3. Proposed Involvement of 9c,11t-CLA and the Calpain-p25-CDK5 Pathway

A potential molecular mechanism was proposed based on findings from the AD model. Nano-PSO, but not native PSO, increased brain levels of the conjugated linoleic acid isomer 9c,11t-CLA, supporting the in vivo conversion of punicic acid to CLA and demonstrating that the nanoformulation improves the brain availability of this metabolite [27]. This CLA isomer was previously shown in vitro to inhibit µ-calpain, and its accumulation in the brain of Nano-PSO-treated 5XFAD mice was associated with reduced p25 levels [27,67]. Since activation of the calpainp25CDK5 pathway is linked to neurodegeneration, tau pathology, and synaptic loss [65,66], these findings suggest that the brain accumulation of 9c,11t-CLA may reduce calpain-related signaling and support a potential mechanistic link between Nano-PSO lipid metabolism and its protective effects on synaptic integrity and amyloid pathology [27].

### 7.4. Anti-Inflammatory Action and Modulation of Neurogenesis

Nano-PSO may promote a more permissive CNS microenvironment by reducing reactive astrogliosis and supporting endogenous neurogenesis. Reduced activation of GFAP-positive astrocytes was observed across several disease models. In the TBI model, Nano-PSO reduced injury-induced astrocyte activation in the cortex when administered either before or after injury [28]. In the ischemia-reperfusion model, treatment reduced the number of GFAP-positive cells in the hippocampal CA1 region by approximately 75% [30]. In the EAE model, Nano-PSO alone significantly reduced gliosis, whereas hMSC transplantation alone had no significant effect; the combined treatment of Nano-PSO and hMSCs produced the greatest reduction among all groups [53]. Together with the reduction in inflammatory markers observed in the CJD model [55], these findings suggest that modulation of the neuroinflammatory response may contribute heavily to the neuroprotective effects of Nano-PSO.

Nano-PSO also supported neuronal survival and neurogenesis across models. Treatment increased neurogenesis in CJD mice [55]. In addition, treatment preserved synaptophysin expression in the CJD model [26], maintained both synaptophysin and SIRT1 expression in the TBI model [28], and reduced neuronal loss in the cortex and dentate gyrus following TBI, as well as in the hippocampal CA1 and CA3 regions following ischemia-reperfusion [28,30]. In the EAE model, staining for the neural progenitor markers Nestin and DCX showed that combined Nano-PSO and hMSC treatment significantly increased the number of endogenous neural progenitor cells in the subventricular and subependymal zones compared to either treatment alone [53].

Interestingly, these neuroprotective effects do not always depend on a reduction in disease-specific protein accumulation. In the CJD model, Nano-PSO did not reduce the accumulation of proteinase K-resistant PrP [26,31,32]. Therefore, its benefits in genetic prion disease may reflect improved neuronal resilience and protection against downstream neurotoxicity rather than direct inhibition of prion aggregation. Alternative explanations include reduced neuroinflammation and preservation of synaptic function, although their contributions have not been directly established. This contrasts with the AD model, where Nano-PSO treatment was explicitly associated with reduced Aβ accumulation, potentially involving the calpain–p25–CDK5 pathway described above [27]. This highlights that while a shared antioxidant and neuroprotective may contribute to the effects of Nano-PSO, its impact on specific protein aggregate pathologies varies by disease context.

### 7.5. Key Mechanisms of Action

Nano-PSO (GranaGard^®^) may act through complementary, multifaceted biological pathways rather than a single target:

- **Systemic Antioxidant & Mitochondrial Protection:** May mitigate oxidative stress, decrease lipid peroxidation, affect mitochondrial proteins (e.g., COX IV-1), and reduces reactive astrogliosis [30,31,34].
- **Pathology-Specific Interventions (e.g., Amyloid Pathology):** Brain accumulation of 9c,11t-CLA was associated with reduced p25 levels. Together with previous in vitro evidence showing that 9c,11t-CLA can inhibit calpain activity and reduce Aβ oligomerization, these findings suggest involvement of the calpain-p25-CDK5 signaling pathway in the observed reduction of amyloid burden [27,67].
- **Pathogen-Independent Neuroprotection:** In genetic models like CJD, Nano-PSO offers strong functional and cognitive benefits without necessarily decreasing pathological protein accumulation (PrP), suggesting that mitochondrial and antioxidant effects may contribute to its main neuroprotective activity, but are unlikely to fully account for the observed therapeutic effects [32,55].
- **Microenvironment & Regenerative Synergy:** Nano-PSO may improve the oxidative and inflammatory CNS microenvironment, thereby supporting the survival, longevity, and functional integration of both endogenous neurogenesis and transplanted stem cells (e.g., neural progenitor and human mesenchymal stem cells) [34,53].

Nano-PSO may overcome some of the pharmacokinetic limitations associated with conventional antioxidant approaches by improving the delivery of potent bioactive lipids directly to the central nervous system [22,23]. Because oxidative stress and mitochondrial dysfunction are shared pathogenic mechanisms across neurological disorders, Nano-PSO has been investigated for its potential neuroprotective effects, including effects on cognitive function, neuronal integrity, and cellular repair [15,20,21]. However, the magnitude and consistency of these effects vary across disease models, and independent validation is needed.

### 7.6. Limitations of the Current Evidence

Preclinical studies have reported beneficial effects of Nano-PSO across several neurological disease models, however, not for all the models we have corresponding human studies, since such studies take years to conclude. Some studies were conducted by students in Prof Gabizon laboratory, which latter become Granalix employees (O.B and K.F), however, the TBI, Parkinson’s disease, and MS pilot studies were conducted by external research groups without company involvement in study design or experimental conduct. The function of company authors in the TBI and MS studies were mainly on suggesting and explanation, but not on planning or discussion of results.

Granalix provided financial support mostly for materials, but had no involvement in the design or conduct of these studies. While potential selection and interpretation biases cannot be excluded completely, studies in additional disease models broaden the evidence base of the results.

Interestingly, the reported effects are not consistent across all formulations, disease models, or outcomes. The smaller 30 nm formulation did not demonstrate beneficial effects in the cited EAE experiment [29], responses in the rotenone model were variable [56], and the preliminary MS pilot study did not show significant improvements in EDSS or SDMT [54]. In addition, we stress that the benefits of GranaGard^®^ are temporary and depends on continues treatment [32], meaning that while beneficial effects, including delayed disease onset or progression, were observed in some models, these findings do not establish a curative effect. Together, these observations suggest that treatment outcomes may depend on formulation characteristics, disease context, and treatment duration.

Clinical studies in CJD and Alzheimer’s disease are ongoing. While their results are not yet available to establish clinical efficacy, no toxicity or any adverse effect was reported. The treated individuals report good acceptance to the treatment, such as less tiredness and increase alert.

Overall, publication bias cannot be excluded completely, and further well-controlled, independently conducted studies are needed to establish the reproducibility, clinical relevance, and long-term efficacy of Nano-PSO.

## 8. Conclusions and Future Perspectives

The accumulated preclinical evidence, together with limited preliminary clinical findings, suggests that Nano-PSO (GranaGard^®^) may represent a potential neuroprotective approach for neurological disorders and acute brain injury. The self-emulsifying nanoformulation we developed shows increased accumulation of the bioactive metabolite of punicic acid (9c,11t-CLA) within the central nervous system [26,49,50]. However, the mechanism of BBB transport remains to be established.

**Figure 1:**
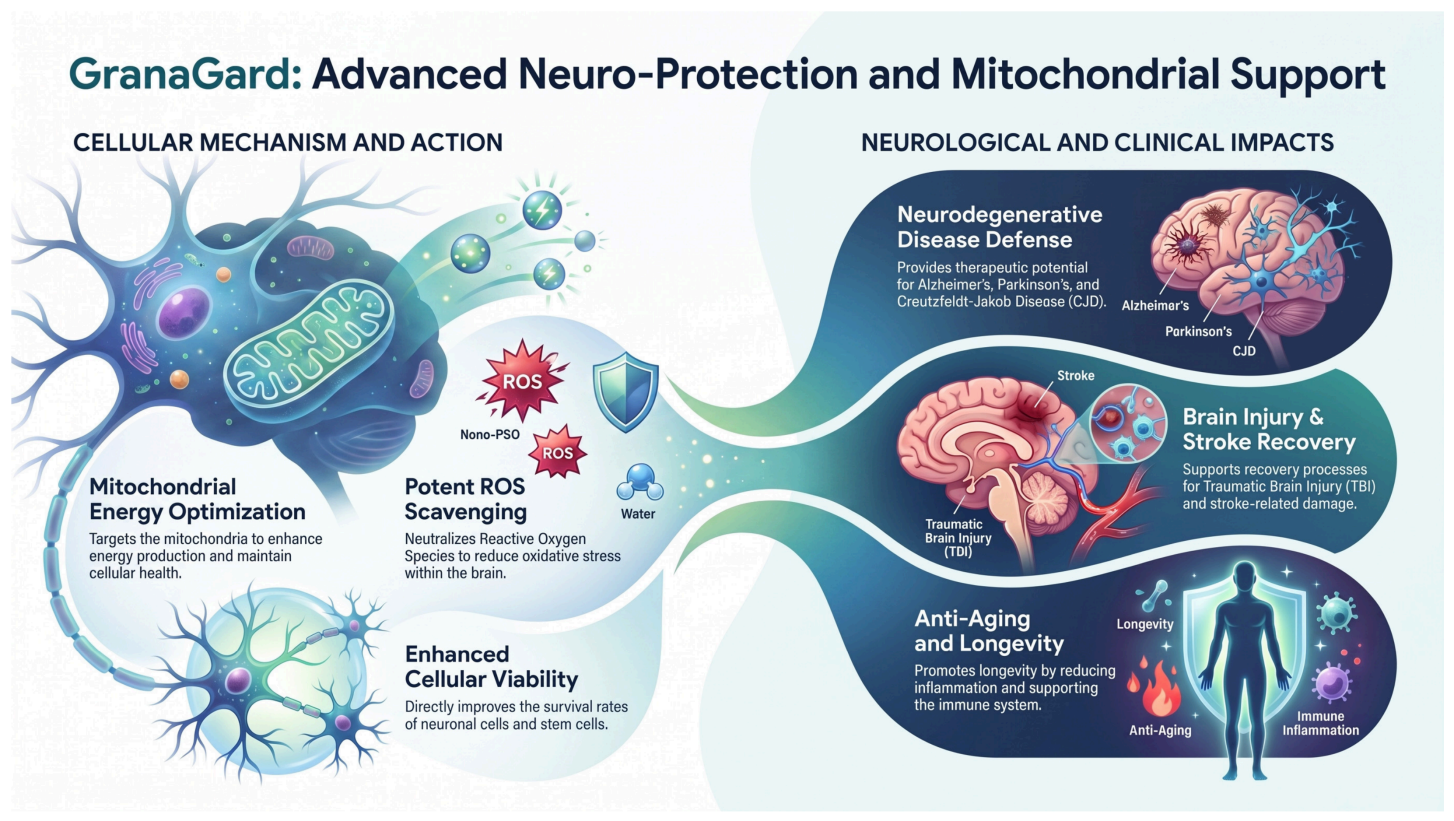
Cellular mechanisms and neurological effects of Nano-PSO (GranaGard^®^). Schematic illustration of the proposed cellular mechanisms and reported effects of Nano-PSO in experimental models of neurological disorders and brain injury. The (**left panel**) illustrates mitochondrial-related changes, reduction of oxidative stress markers, and potential effects on neuronal viability. The (**right panel**) summarizes findings from preclinical studies involving neurodegenerative diseases, including Alzheimer’s disease, Parkinson’s disease, and prion disease, as well as experimental models of traumatic brain injury and cerebral ischemia. Aging-related outcomes are also illustrated, although general anti-aging and longevity effects have not been established. The proposed mechanisms are based on findings from different experimental models and should not be interpreted as evidence of direct mitochondrial targeting or ROS scavenging in the brain. Clinical evidence remains limited, and the findings do not establish therapeutic efficacy in humans across the conditions illustrated. This picture was generated by Notebook LM, Gemini.

Rather than acting on a single pathological target, Nano-PSO has been associated with effects on multiple biological pathways across several preclinical models of neurodegenerative diseases, cerebral ischemia, and traumatic brain injury (Figure 1). Furthermore, the magnitude and consistency of the reported effects vary across models, and independent replication is needed to establish their reproducibility.

## Figures and Tables

**Table 1:** Summary of Preclinical and Available Clinical Studies Evaluating the Neuroprotective Effects of Nano-PSO (GranaGard^®^) Across Neurological Disease Models.

Disease/Condition	Model/Study Design	Main Pathological Features	Key Findings	Proposed Mechanisms of Action
**Multiple Sclerosis (MS)**	EAE mouse model	Neuroinflammation; demyelination; oxidative stress; neurological dysfunction.	Reduced clinical disease severity. Early treatment delayed onset and reduced disease burden, whereas delayed treatment reduced clinical scores without changing disease kinetics. Demyelination and oxidized lipid accumulation were also reduced [29]. A smaller 30 nm formulation was ineffective [29].	Reduced lipid peroxidation and demyelination; efficacy depended on nanoemulsion droplet size, with 180 nm droplets being effective.
**stem cell therapy/multiple Sclerosis (MS)**	EAE mouse model with hMSC transplantation	Neuroinflammation; graft rejection; impaired endogenous repair.	Nano-PSO combined with hMSC transplantation-maintained protection, increased endogenous neurogenesis, reduced astrogliosis, and prolonged detection of transplanted human cells [53].	Reduced oxidative and inflammatory environment prolonged graft survival; suppression of astrogliosis (GFAP); enhanced endogenous neurogenesis (DCX, Nestin).
**Multiple Sclerosis (MS), clinical**	Human: Randomized, double-blind, crossover pilot study (*n* = 30; 22 completed); add—on to immunomodulatory therapy	Cognitive impairment; neurological disability.	Improved verbal learning (CVLT-II) and overall cognitive z-scores. A trend toward improved visuospatial memory was observed, with no significant change in SDMT or EDSS [54]. Preliminary results suggest GranaGard^®^ reduces NfL levels.	Not directly assessed.
**Prion Disease (gCJD)**	TgMHu2ME199K transgenic mouse model	PrP accumulation; mitochondrial dysfunction; oxidative stress; neuroinflammation; synaptic loss; impaired neurogenesis.; GAG accumulation.	Delayed disease progression, reduced lipid oxidation and neuronal loss, preserved synaptophysin, and increased hippocampal neurogenesis. PrP accumulation was not reduced [26]. Continuous treatment prolonged survival. At 18 months, 50% of treated mice had not reached terminal disease, compared to none of the untreated mice. Treatment discontinuation led to rapid deterioration; GAG accumulation was reduced [32].	Clinical benefit observed without reduced PrP accumulation; downstream protective mechanisms proposed, not established; benefit depended on continued treatment.
**Aging-related markers in a prion model**	GranaGard^®^ versus metformin in TgMHu2ME199K mice	Mitochondrial dysfunction; oxidative stress; metabolic decline; neurodegeneration	Both treatments (Nano-PSO and Metformin) affected AMPK, COX IV-1, Nrf2/HIF-1α, IGF-1, inflammatory markers, and neural stem-cell generation; however, only Nano-PSO delayed clinical disease progression [55].	Shared effects on aging-related pathways alone do not fully explain Nano-PSO clinical benefits; additional specific neuroprotective mechanisms are likely involved.
**stem cell therapy/prion disease (gCJD)**	TgMHu2ME199K transgenic mouse model with NPC transplantation	Symptomatic neurodegeneration; low graft survival.	Nano-PSO combined with NPC transplantation extended disease arrest from 30–40 days to 70–80 days, prolonged overall survival, increased endogenous neurogenesis, and maintained transplanted NPC viability and migration longer [33,34].	Antioxidant and anti-inflammatory effects improved graft survival and enhanced endogenous neurogenesis.
**Alzheimer’s Disease (AD)**	5XFAD mouse model	Aβ accumulation; amyloid plaque formation; oxidative stress; mitochondrial dysfunction; synaptic impairment; cognitive decline.	Preserved spatial memory and habituation, reduced intra- and extracellular Aβ and amyloid plaque burden, restored COX IV-1, increased brain 9c,11-CLA, and reduced p25 levels [27].	Antioxidant and mitochondrial protection (COX IV-1); punicic acid conversion to 9c,11t-CLA with proposed inhibition of the calpain–p25–CDK5 pathway, reducing amyloid pathology and preserving synapses.
**Acute Brain Injury (TBI)**	Mouse traumatic brain injury (TBI) model	Oxidative stress; mitochondrial dysfunction; astrogliosis; neuronal loss; cognitive impairment.	Pre- and post-injury treatment improved visual and spatial memory, reduced cortical and hippocampal neuronal loss and astrogliosis, and restored COX IV-1. Post-injury treatment also elevated SIRT1 and synaptophysin. Pre-injury treatment was more effective [28].	Antioxidant; anti-inflammatory effects (GFAP); mitochondrial protein markers (COX IV-1, SIRT1); and synaptic protective effects (synaptophysin).
**Acute Brain Injury (Stroke)**	Rat model of cerebral ischemia-reperfusion injury	Oxidative stress; BBB disruption; astrogliosis; hippocampal neuronal loss; motor and cognitive deficits.	Improved locomotor recovery and spatial working memory; reduced hippocampal neuronal loss, astrogliosis, lipid peroxidation, and nitric oxide metabolites. Pre-treatment also preserved BBB integrity and was more effective overall [30].	Antioxidant activity (reduced MDA and nitric oxide metabolites); preserved BBB integrity (distinct from transport of the formulation across the BBB); anti-inflammatory effects (GFAP); hippocampal neuroprotection (CA1, CA3).
**Parkinson’s Disease (PD)**	Rotenone (ROT)-induced rat model	Mitochondrial complex I inhibition; oxidative stress; dopaminergic neuronal damage; α-synuclein alterations; motor dysfunction.	Moderate improvements in motor, neuronal, dopaminergic, oxidative and transcriptional parameters. Preventive treatment generally gave greater effects on motor performance, dopaminergic neuron preservation and lipid peroxidation. Responses varied considerably between animals, and not all parameters were restored [56].	Antioxidant effect (reduced MDA); mitochondrial protection (proposed, not directly measured); reduced α-synuclein accumulation; possible serotonergic compensatory support (proposed).

**Abbreviations:** AMPK, AMP-activated protein kinase; Aβ, amyloid-beta; CDK5, cyclin-dependent kinase 5; CLA, conjugated linoleic acid; COX IV-1, cytochrome c oxidase subunit IV-1; DCX, doublecortin; EAE, experimental autoimmune encephalomyelitis; EDSS, Expanded Disability Status Scale; GAG, glycosaminoglycans; GFAP, glial fibrillary acidic protein; HIF-1α, hypoxia-inducible factor 1-alpha; hMSC, human mesenchymal stem cells; IGF-1, insulin-like growth factor 1; MDA, malondialdehyde; NfL, neurofilament light chain; NPC, neural progenitor cells; Nrf2, nuclear factor erythroid 2-related factor 2; PrP, prion protein; SDMT, Symbol Digit Modalities Test; SIRT1, sirtuin 1.

## Data Availability

No new data were created or analyzed in this study. Data sharing is not applicable to this article.

## Abbreviations

Nano-PSO, nanoformulated pomegranate seed oil; ROS, reactive oxygen species; TBI, traumatic brain injury.
